# Assessment of disturbed glucose metabolism and surrogate measures of insulin sensitivity in obese children and adolescents

**DOI:** 10.1038/s41387-017-0004-y

**Published:** 2017-12-14

**Authors:** Christian L Roth, Clinton Elfers, Christiane S Hampe

**Affiliations:** 1 Center for Integrative Brain Research, Seattle Children’s Hospital and Research Institute, Endocrine Division, Seattle, WA 98101 USA; 2 Division of Metabolism, Endocrinology and Nutrition, Department of Medicine, University of Washington, Seattle, WA 98109 USA

## Abstract

**Background:**

With the rising prevalence of obesity and type 2 diabetes (T2D) in obese children, it is becoming imperative to detect disturbed glucose metabolism as early as possible in order to prevent T2D development.

**Subjects/Methods:**

Cross-sectional study of 92 obese children (median age 11.7 years, 51% female) and 7 lean children (median age 11.4 years, 57% female) who underwent an oral glucose tolerance test (OGTT) in a tertiary pediatric care center. Glucose tolerance was assessed and different indices for β-cell function, insulin sensitivity and insulin secretion were calculated.

**Results:**

Nineteen obese children were identified with prediabetes (PD, 12 impaired glucose tolerance, 4 increased fasting glucose and 3 combined). Compared with the 73 obese children with normal glucose tolerance (nGT), subjects with PD had higher insulin resistance, but lower insulin sensitivity and β-cell function, although their glycated hemoglobin (HbA_1c_) levels were comparable. The Whole Body Insulin Sensitivity Index (WBISI) and β-cell function by Insulin Secretion-Sensitivity Index-2 (ISSI-2) strongly correlated with the OGTT glucose area under the curve 0–120 min (r = 0.392, *p* < 0.0002; r = 0.547, *p* < 0.0001, respectively). When testing the relation between early insulin response during OGTT by insulinogenic index and insulin sensitivity assessed by WBISI, a hyperbolic relationship between insulin secretion and insulin sensitivity was found. The calculated disposition index was lower in subjects with PD vs. nGT (median 459 vs. 792, *p* = 0.004). We identified the OGTT 30-min/120-min insulin ratio as a simple marker, which is significantly lower in obese children with vs. without PD (median 0.87 vs. 1.29, *p* = 0.021) and which has a better sensitivity and specificity for detecting PD than HbA_1c_ among obese children.

**Conclusions:**

Children with identified PD had changes of several markers for β-cell function, insulin sensitivity and resistance before changes in HbA_1c_ occurred. The lower disposition index indicates that these children have already inadequate β-cell compensation for the degree of insulin resistance.

## Introduction

In the last 10 years, the incidence of type 2 diabetes (T2D) has increased from <3 to 10–45% of new-onset diabetes in youth, and every year ~5000 youth are diagnosed with T2D in the United States alone^1–4^. Among adolescents in the US aged from 12 to 19 years, the prevalence of prediabetes or diabetes increased from 9% to 23% from 1999-2000 to 2007-2008, with a concomitant dramatic increase of other risk factors for cardiovascular disease^5^. Obesity-induced inflammation, endothelial dysfunction, atherosclerosis, and myocardial ischemia are key features of T2D^6^. Pediatric obesity is a risk factor for insulin resistance and T2D, and it is crucial to identify subjects who are at risk for developing T2D. Per recommendation of the American Diabetes Association (ADA), testing for prediabetes (PD) defined by impaired fasting glucose (IFG) and/or impaired glucose tolerance (IGT) should be considered in children and adolescents who are overweight or obese (Ob) and have two or more additional risk factors for diabetes^7^.

Diabetes, IFG and IGT are defined by glucose measures in the fasting state, as well as at 2-h during a standardized oral glucose tolerance test (OGTT). However, recent evidence shows that glucose and hemoglobin A_1c_ (HbA_1c_) levels rise already before the clinical diagnosis of diabetes^7, 8^, allowing diagnosis of PD defined by IFG or IGT well before the onset of diabetes mellitus. According to recent ADA guidelines, to test for PD, fasting plasma glucose, OGTT 2-h blood glucose (BG) and HbA_1c_ levels, are considered as equally appropriate^7^. However, the benefit of including elevated HbA_1c_ levels in the identification of individuals with PD or diabetes is still debated, particularly in children^9–11^.

T2D is characterized by the combination of both insulin resistance and development of β-cell dysfunction^12^. Many metabolic markers and indices, most of them calculated by fasting and stimulated levels of glucose and insulin, have been used in previous studies to assess β-cell function, insulin sensitivity and resistance. Hyperinsulinemic–euglycemic clamp studies are considered as the gold standard for assessing insulin sensitivity and resistance^13^. In children, calculated indices based on fasting or stimulated measures of plasma glucose and insulin are more feasible alternatives, which correspond well with insulin sensitivity based on hyperinsulinemic–euglycemic clamp studies^14–16^. Insulin resistance is also associated with hypertriglyceridemia and the triglyceride glucose index (TyG) has been proposed as a measure for insulin resistance without measuring insulin levels^17, 18^. In this study, we analyzed glucose tolerance and different parameters of glucose tolerance, as well as insulin secretion and sensitivity. The goal was to determine which surrogate markers for β-cell function and insulin sensitivity correspondent well with glucose tolerance, and which measures are feasible for predicting PD in this cohort of Ob children.

## Subjects and methods

### Study population

Our study cohort consisted of 92 Ob children and 7 lean children of comparable age, sex, ethnicity (Caucasian) and pubertal stage (Table 1). The Ob children were consecutively seen and tested at the pediatric obesity clinic. All Ob children had a body mass index (BMI) > 97th percentile for age and sex. Patients with syndromal obesity, history for brain tumors, malignancies or underlying chronic disorders were excluded from the study. We included as controls lean subjects with a BMI between the 10th and 90th percentile for age and sex. These control subjects were healthy volunteers recruited at the pediatric outpatient clinic and subjects in whom an endocrine disorder could be excluded. All participants were recruited in the area of Bonn, Germany. Both groups were presented and examined at the Department of Pediatrics, University of Bonn, a tertiary care center. None of the children suffered from previously diagnosed T2D, hypothyroidism, chronic disorders or were taking prescription medication. All underwent metabolic testing including an OGTT with venous blood sampling. Study protocols were approved by the local standing committee for clinical studies and the committee on ethical practice at the University of Bonn, Germany, as well as by the institutional review board at Seattle Children’s Research Institute, Seattle, USA. Written parental consent and/or patient assent was obtained and investigations were conducted according to the principles expressed in the Declaration of Helsinki.

**Table 1:** Characteristics of 99 studied children with normal weight (NW) or obesity (OB) with normal glucose tolerance (nGT) and prediabetes (PD)

	**NW**	**Ob nGT**	**Ob PD**	*p***-Value Ob nGT** vs. **PD** (unadjusted^a^/adjusted^b^)
Age	11.4 (10.1, 14.6)	11.6 (7.8, 13.9)	11.89 (9.4, 13.5)	0.797/na
Gender (m/f)	3/4	35/38	10/9	0.799/na
Puberty (pre/pubertal)	3/4	40/33	11/8	1.000/na
SDS-BMI	−1.04 (−2.69, 0.54)	2.90 (2.55, 3.40)	2.90 (2.70, 3.10)	0.725/0.454
BG fasting (mg/dL)	89 (76, 92)	83 (76, 88)	93 (80, 100)	0.002/0.002
Insulin fasting (mU/L)	2.0 (2.0, 4.5)	10.5 (6.1, 17.3)	12.0 (6.2, 20.1)	0.589/0.003
WBISI	12.55 (9.08, 16.86)	3.43 (2.27, 6.35)	2.65 (1.29, 4.10)	0.0211/<0.0001
ISSI2	3.11 (2.55, 3.65)	2.47 (2.13, 2.84)	1.72 (1.56, 1.92)	0.0001/0.0001
IGI	65.7 (56.0, 267.7)	237.5 (118.1, 388.5)	249.5 (143.2, 304.1)	0.886/0.565
HOMA-IR	0.45 (0.40, 1.03)	2.20 (1.29, 3.61)	2.45 (1.65, 4.04)	0.237/0.001
HOMA-%B	44.4 (28.7, 57.6)	210.9 (85.4, 338.4)	134.5 (72.2, 280.6)	0.590/0.115
QUICKI	0.443 (0.381, 0.454)	0.339 (0.316, 0.368)	0.334 (0.311, 0.396)	0.237/<0.0001
FGIR	40.0 (20.7, 44.5)	7.5 (4.5, 14.3)	8.2 (4.3, 15.9)	0.927/<0.0001
TyG	na	8.24 (8.05, 8.60)	8.62 (8.19, 8.82)	0.057/0.022
HbA1c	na	5.3 (5.1, 5.5)	5.4 (5.2, 5.7)	0.187/0.623

Data presented as median (25%, 75%). For comparisons between the Ob nGT and PD groups:

a.*p*-Value from Mann–Whitney *U*-test for differences of a continuous variable or from Fisher’s exact probability test for a categorical variable

b.*p-*Value for differences of a continuous variable adjusting for age, puberty and sex using multivariable regression analyses; puberty and sex are categorical variables

### Anthropometric data

Obesity status was defined exceeding the 97th percentile according to population-based standards set by the German Working Group on Obesity in Childhood and Adolescence (AGA)^19^. Height was measured to the nearest centimeter using a rigid stadiometer, and weight was measured in underwear to the nearest 0.1 kg using a calibrated balance scale. The standard deviation scores (SDS), SDS-height, SDS-weight, and SDS-BMI, were calculated according to German percentiles as described in detail previously^19–21^. Pubertal developmental stages were assessed by a pediatric endocrinologist using the standards of Marshall and Tanner^22, 23^.

### OGTT and biochemical parameters

Patients fasted overnight (at least 12 h). Blood sampling was performed in the fasting state at 0800 hours and glucose (1.75 g/kg body weight, max dose 75 g, Dextro O.G.T.^®^; Roche, Grenzach-Wyhlen, Germany) was administered orally between 0800 and 0900 hours. Blood was drawn at 0, 30, 60, 90, 120 and 180-min post glucose administration using an indwelling intravenous line into pre-chilled tubes to determine BG and insulin levels during the OGTT^24^. Samples were immediately centrifuged at 4 °C, aliquoted, and stored at −80 °C. Serum insulin concentrations were measured by microparticle enhanced immunometric assay (MEIA^TM^, Abbott, Wiesbaden, Germany). Specimens for quantification of plasma glucose were collected in tubes containing a glucolytic inhibitor (Sarstedt, Nümbrecht Germany) and plasma glucose levels were determined by colorimetric examination using a Vitros^TM^ analyzer (Ortho Clinical Diagnostics, Neckargmϋnd, Germany). Intra- and inter-assay measurements of the coefficient of variation were <8%. Glucose and insulin areas under the curve (AUC) were calculated from 0 to 120 min of the OGTT using the trapezoidal method. High-density lipoprotein cholesterol, low-density lipoprotein cholesterol and triglycerides were measured by standard techniques. The TyG is a surrogate maker of insulin resistance calculated as the product of fasting triglycerides and glucose^17, 18^. HbA_1c_ was determined in EDTA-whole blood using standard techniques at the Clinical Chemistry laboratory, University of Bonn. Cut-off values for fasting glucose and 120-min glucose for PD and diabetes were set according to the guidelines provided by the AGA^19^. Ethylenediaminetetraacetic acid (EDTA)

The following indeces were used for the determination of insulin resistance and sensitivity: Pancreatic *β-cell function* was assessed by calculating the homeostasis model assessment (HOMA) derived β-cell function (HOMA-%B) index, defined as fasting insulin mU/L × 20)/(fasting glucose mmol/L – 3.5) and the insulinogenic index (IGI), which is calculated by the ratio of the increase of the insulin level to the increase of the glucose level during 0–30 min of the OGTT^25, 26^*. Insulin resistance* was assessed from fasting glucose and insulin concentrations using the formula for the HOMA of insulin resistance, HOMA-IR =insulin [mU/L] × glucose [mmol/L])/22.5^26^. *Insulin sensitivity* was estimated by calculating the fasting glucose insulin ratio (FGIR), by QUantitative Insulin sensitivity ChecK Index (QUICKI =1/[log insulin (mU/L) + log baseline glucose (mg/dL)) and by Matsuda Whole Body Insulin Sensitivity Index (WBISI) =10 000 /(fasting glucose × fasting insulin × mean glucose concentration × mean insulin concentration)^1/2^, which encompasses both hepatic and peripheral tissue insulin sensitivity^14^. *Insulin resistance-adjusted β-cell function* was established by the Insulin Secretion-Sensitivity Index-2 (ISSI-2), which was calculated as AUC_ins0-120_/AUC_gluc0-120_ × WBISI^27, 28^. We examined the relationship between insulin secretion and insulin sensitivity by testing whether early insulin response during the OGTT (IGI) and a surrogate measure of insulin sensitivity (1/fasting insulin) to calculate a disposition index, which provides a measure of β-cell function adjusted for insulin sensitivity and has been shown to be predictive of development of diabetes^29^. In addition, we tested the relationship between IGI and WBISI as the alternative surrogate for insulin sensitivity. Finally, we calculated the resulting disposition index for each relation as IGI × 1/fasting insulin^29^, which was compared with IGI × WBISI.

### Statistical analysis

Linear mixed-effects models were used for analysis of insulin and glucose measures during OGTT. Post-hoc pairwise comparisons of marginal linear predictions were made using a Bonferroni post-test at each time point. Simple regression was used for the comparison of two continuous variables, whereas multivariable regression analyses were used to adjust for age, sex and puberty; sex and puberty were treated as categorical variables, age was treated as continuous variable. For two group comparisons, we used Student’s *t*-test for normally distributed values and Mann–Whitney *U*-test as a non-parametric test or Fisher's exact probability test as indicated. For multiple group comparisons of normally distributed data, a one-way analysis of variance (ANOVA) was used for comparison of means with a Bonferroni post-test for multiple pairwise comparisons. Similarly, for multiple group comparisons with non-parametric data a Kruskal–Wallis test was used to compare medians with Dunn’s post-test for multiple pairwise comparisons. All statistical testing was two-sided, and *p*-values <0.05 were considered statistically significant. Statistical analyses were performed using the Prism® program (GraphPad, San Diego, CA, USA) and STATA (StataCorp LLC, College Station, TX, USA).

## Results

A total of 99 pediatric subjects (48 males and 51 females) were enrolled in this study, 45 of which were pubertal (see Table 1). All of the seven lean subjects had normal glucose tolerance (nGT). Of the 92 Ob subjects, 73 (79%) had normal fasting glucose and nGT, whereas four had IFG (100–125 mg/dL), 12 had IGT (OGTT 2-h BG >140 mg/dL) but normal fasting glucose and an additional three subjects had IFG and IGT, resulting in a total number of 19 subjects with PD.

### Insulin sensitivity and secretion in Ob children with vs. without PD

Upon analyzing different indices related to insulin sensitivity and secretion based on fasting levels of insulin and glucose (HOMA-R, HOMA-%B, FGIR and QUICKI), OGTT-derived indices (WBISI, ISSI-2 and IGI) and other markers of insulin resistance and glucose tolerance (TyG and HbA_1c_), the two Ob groups showed significantly different results for most of the parameters before and after adjusting for age, puberty and sex, but not for adjusted results for HOMA-%B, IGI and HbA_1c_ (Table 1). Regarding results for HOMA-%B, there were no correlations with well-established markers of insulin secretion, such as IGI and ISSI-2. HOMA-%B results were dependent on the degree of insulin resistance instead (HOMA-%B vs. WBISI (Ln transformed data) r = 0.748, *p* < 0.0001; HOMA-%B vs. ISSI-2 r = 0.275, *p* = 0.174). Therefore, HOMA-%B was not included in further testing.

### Analysis of glucose levels and insulin secretion during OGTT

**Fig. 1:**
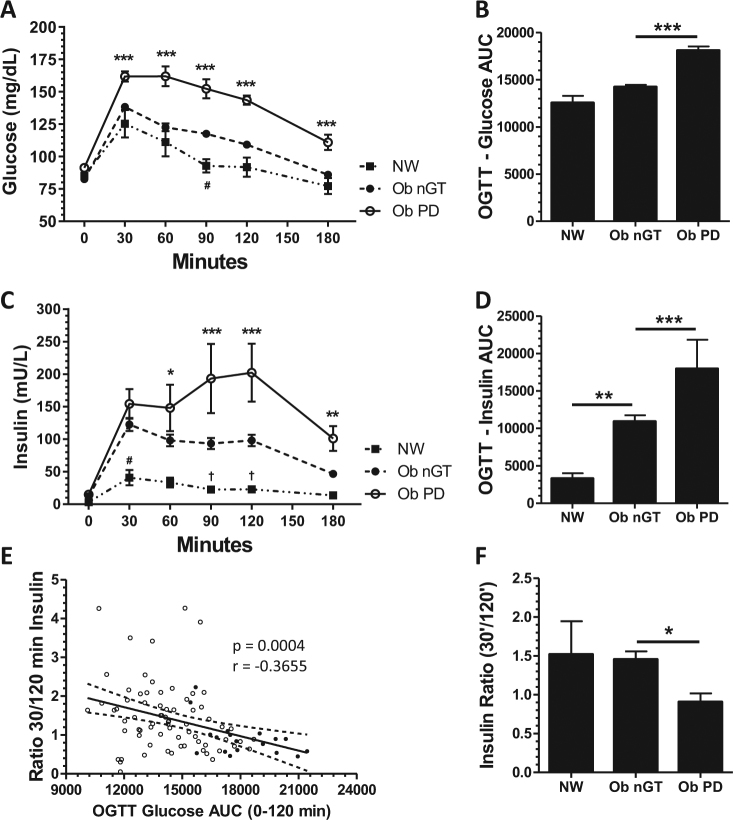
Glucose and insulin levels following the oral glucose load in Ob subjects with PD compared with Ob subjects with nGT and lean controls by a linear mixed model and pairwise comparisons of marginal linear predictions. NW vs. Ob nGT: ^†^*p* < 0.10, ^#^*p* < 0.05; Ob nGT vs. Ob PD: *p < 0.05, **p < 0.01, ***p < 0.001 **a**, **c**, adjusted for multiple comparisons. The glucose AUC 0–120 min was calculated and compared between the three groups by a one-way ANOVA and Bonferroni’s post-test. *** *p* < 0.001 **b**. Insulin 0–120 min AUC was compared using a Kruskal–Wallis test and Dunn’s post-test. **p < 0.01, ***p < 0.001 **d**. Spearman correlation between insulin ratio 30/120 min and OGTT-AUC_Gluc0-120_ showing values of Ob subjects with nGT (open circles) and PD (closed circles) **e**. Ratio of insulin levels at 30 min over 120 min comparing Ob subjects with PD with Ob subjects with nGT and lean controls by a Kruskal-Wallis test and Dunn’s post-test. *p < 0.05 **f**

Glucose levels following the oral glucose load were similar between control subjects and Ob subjects with nGT except for the 90-min time point, whereas subjects with PD had higher glucose levels during the entire duration of the test compared with the other two groups (Figs. 1a, b). However, during the OGTT, Ob subjects with nGT had significantly higher insulin levels at 30 min and here higher in trend at 90 and 120 min when compared with control subjects (Figs. 1c, d), although glucose levels showed only a slightly difference between the two groups at 90 min. Although Ob subjects with nGT and control subjects demonstrated a downward trend in insulin levels from 30 to 120 min, insulin levels increased from 30 to 120 min in Ob subjects with PD (Figs. 1c, d). A higher ratio of insulin levels at 30 min over 120 min correlated with lower OGTT-AUC_Gluc0-120_ indicating a better glucose tolerance (Fig. 1e). Consequently, the ratio of insulin levels at 30 min over 120 min was significantly lower in Ob subjects with PD compared with Ob subjects with nGT and lean controls, whereas there were no significant differences between the latter two groups (Fig. 1f).

### Analyses based on glucose area under the curve during OGTT

**Fig. 2:**
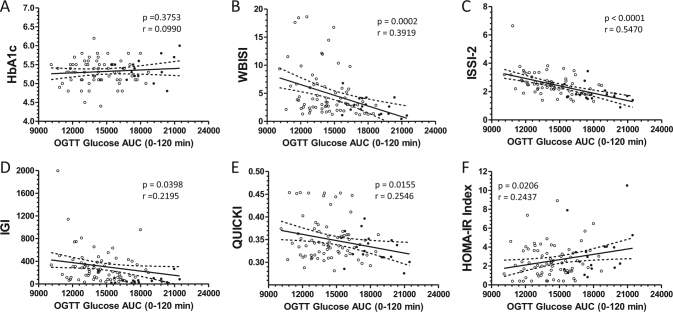
Relationship between glucose control during OGTT assessed by OGTT-AUC_Gluc0-120_, and measures of glucose tolerance **a**, insulin sensitivity **b**, **e**, insulin secretion **c**, **d**, as well as insulin resistance **f**, showing values of Ob subjects with nGT (open circles) and PD (closed circles)

In 37% (34/92) of Ob subjects, glucose levels were lower at 90 min than at 120 min showing an N-shaped biphasic curve. Twenty percent (7/34) of these subjects were classified as PD. To further analyze the risk for glucose intolerance and diabetes, we calculated the OGTT-AUC_gluc0-120_, which correlated significantly with the glucose level at 120 min (r = 0.7247, *p* < 0.0001), WBISI, ISSI-2, IGI, and fasting indices QUICKI and HOMA-IR, but not with TyG (r = 0.1584, *p* = 0.1660), and HbA_1c_ (Figs. 2a-f). Of the OGTT-derived measures, WBISI is considered as gold standard, which correlates well with insulin sensitivity calculated from hyperinsulinemic clamp results^14, 30^. We tested whether WBISI correlated with indices based on fasting measures and found a highly significant correlation between WBISI and QUICKI (r = 0.905, *p* < 0.0001). After adjusting for age, puberty and sex, OGTT-AUC_gluc0-120_ correlated significantly with WBISI, ISSI-2 and IGI. OGTT glucose_120_ correlated significantly with WBISI and ISSI-2. OGTT insulin_120_ correlated significantly with WBISI, ISSI-2, IGI, HOMA-IR, QUICKI, FGIR, TyG and HbA_1c_ (Table 2). When dividing the calculated OGTT-AUC_gluc 0-120_ into tertiles, we found that WBISI and ISSI-2 were significantly lower comparing the upper vs. lower tertiles (Suppl Fig. 1).

**Table 2:** Relationships between measures of glucose metabolism and surrogate markers for insulin sensitivity and resistance

	**120**-min **insulin**	**120-m**in **glucose**	**OGTT glucose AUC**
	Unadjusted/adjusted	Unadjusted/adjusted	Unadjusted/adjusted
120-min glucose	<0.0001/<0.0001	na	<0.0001/<0.0001
120-min insulin	na	<0.0001/0.001	<0.0001/<0.0001
WBISI	<0.0001/0.0001	0.003/0.035	0.0002/0.005
ISSI2	0.028/0.029	<0.0001/0.0001	<0.0001/<0.0001
IGI	0.053/0.019	0.109/0.553	0.040/0.034
HOMA-IR	<0.0001/<0.0001	0.052/0.317	0.021/0.090
QUICKI	<0.0001/<0.0001	0.045/0.339	0.016/0.095
FGIR	0.0001/0.001	0.153/0.678	0.089/0.180
TyG	0.005/0.011	0.386/0.690	0.166/0.259
HbA1c	0.095/0.030	0.1140/0.524	0.375/0.305

Unadjusted *p*-values were calculated by simple regression analyses. Adjusted *p*-values were calculated by multivariable regression analyses with age, puberty and sex as independent variables

### Analysis of the disposition index based on OGTT-derived vs. fasting measures

**Fig. 3:**
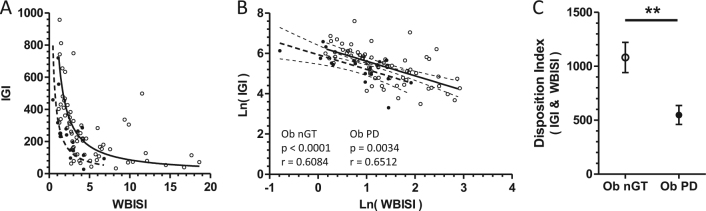
Analysis of the disposition index based on OGTT-derived vs. fasting measures. OGTT disposition index calculated IGI and WBISI resulted in significantly lower results for Ob children with PD compared with Ob children without PD **a**–**c**, showing values of Ob subjects with nGT (open circles) and PD (closed circles). **P < 0.01 by Mann–Whitney *U*-test

When comparing the disposition indices, a hyperbolic relationship between measures of insulin sensitivity and β-cell function could be demonstrated. Using IGI as an assessment of β–cell function and 1/fasting insulin for insulin sensitivity, the results of OGTT disposition index of Ob subjects with nGT significantly differed from those with PD (data not shown, median/interquartile range Ob nGT: 3.163 (2.191, 4.852), Ob PD: 2.063 (1.603, 2.556), *p* = 0.0225). However, a clearer separation between Ob subjects with vs. without PD was found for the disposition index calculated from OGTT-derived WBISI for insulin sensitivity and IGI for insulin secretion, as significantly lower results for Ob children with PD were found compared with Ob children without PD (Fig. 3).

### Comparison of different surrogate markers for disturbed glucose metabolism

Using the recommended threshold for HbA_1c_ ≥5.7% to detect PD^7^, our cohort showed a low sensitivity for HbA_1c_ of 23.5% and specificity of 86.6% for detecting PD. After lowering the threshold for HbA_1c_ to 5.4% as discussed previously^7^, the sensitivity increased to 58.8% and specificity changed to 55.2%. We also tested HOMA-IR 95th and 75th percentiles using the published age- and sex-specific thresholds from Allard et al.^31^, and compared the sensitivity and specificity for detecting PD with thresholds we determined in our study for using WBISI (<2.25), disposition index (IGI × WBISI <700), OGTT insulin_120_ (>120 mU/L) and the ratio OGTT insulin 30 min/120 min (<1.2). These measures and indices showed significantly higher sensitivity to detect PD than the recommended HbA_1c_ ≥5.7% (Table 3).

**Table 3:** Sensitivity and specificity of surrogates for predicting prediabetes

	HbA1c (≥5.4%)	HbA1c (≥5.7%)	HOMA-IR (>75%)	HOMA-IR (>95%)	WBISI (≤2.25)	WBISI and IGI DI (<700)	Ins120 (>120 mU/L)	Ins 30/120 Ratio (<1.2)
Sensitivity	58.8%	23.5%	73.7%	47.4%	44.4%	77.8%	66.7%	88.9%
Specificity	55.2%	86.6%	34.2%	64.4%	72.6%	60.0%	69.0%	54.8%
Positive predictive value (PPV)	21.3%	6.5%	35.9%	16.1%	13.1%	25.0%	19.7%	28.6%
Negative predictive value (NPV)	84.1%	81.7%	83.3%	82.5%	84.1%	91.3%	89.1%	95.2%

## Discussion

In this study of 92 Ob children, 19 (20%) subjects were diagnosed with PD. These numbers are in agreement with previous studies in Ob children and adolescents^32, 33^, but are lower than the frequency of PD reported in African-American and Hispanic Ob children and adolescents^34, 35^. Our cohort consists of Caucasian children only, which may account for the relatively lower prevalence rate of PD compared with these multi-ethnical cohorts. Insulin secretion patterns during an OGTT have not been the focus of much prior research in children. In order to estimate the risk for diabetes development, we performed a comprehensive analysis of different surrogate measures for disturbed glucose metabolism. First, we used standard criteria, for example, fasting and OGTT 2-h glucose levels for the assessment of PD (either IFG or IGT). Defining IGT based on OGTT, 2-h glucose levels has some limitations such as poor reproducibility and additional incretin effects, which are well described in the literature^36^, but remains a common practice. Compared with those without PD, Ob children with PD showed decreased insulin resistance-adjusted β-cell function as assessed by ISSI-2, reduced insulin sensitivity expressed as WBISI and QUICKI, and reduced disposition index calculated by IGI and WBISI. Furthermore, we found that in patients with PD, OGTT insulin levels at 120 min were markedly higher compared with subjects without PD. This was demonstrated by a decreased ratio of OGTT insulin ratio at 30 min over 120 min, which in our cohort showed best combined sensitivity and specificity for detecting PD among the different glucose metabolism markers.

In 34 of the 92 Ob subjects, we found that the OGTT glucose levels at 90 min were lower than at 120 min, indicating a biphasic glucose response, which is known to be associated with a better glucose tolerance and higher insulin sensitivity than a monophasic glucose response^36^ and suggests that elevated 120 min glucose values are caused by counter regulation rather than the degree of IGT. Therefore, we also analyzed the different surrogate markers in the context of the OGTT-AUC_gluc0-120_ as a continuous measure under the assumption that the OGTT-AUC_gluc0-120_ correlates with the risk for PD, diabetes and obesity-related cardiovascular disease^37, 38^. Indeed, higher insulin resistance and higher glucose excursions were found to be strongly correlated with oxidative stress and higher risk for cardiovascular disease even among subjects with nGT^39–41^. We found that WBISI, ISSI-2 and IGI correlated significantly with the OGTT-AUC_gluc0-120_, whereas there were no significant correlations between OGTT-AUC_gluc0-120_ and fasting blood-derived indices as well as TyG or HbA_1c_.

The hyperinsulinemic–euglycemic clamp and the hyperglycemic clamp are regarded as the best standard methods for measuring insulin sensitivity and pancreatic β-cell function, respectively^42^. However, as these procedures are invasive and labor intensive, we analyzed fasting blood and OGTT-derived measures as simple surrogates that have been shown to correlate with the clamp procedures. Among those, WBISI was found to represent a good estimate for clamp-derived insulin sensitivity^30^. We found that among the fasting blood-derived measures, QUICKI correlated best with WBISI. TyG, a marker used for assessment of insulin resistance and risk for cardiovascular disease^17, 18^, and HbA_1c_, a recommended screening marker for PD and diabetes^7^, did not correspond with WBISI, OGTT-AUC_Gluc0-120_ or OGTT glucose_120min_. In contrast, a significant negative correlation between OGTT-AUC_Gluc0-120_ and IGI indicated a reduced early phase insulin response and therefore declining β-cell function.

It is known that in Ob subjects, a progressive decline of the first-phase insulin response and alteration of liver insulin sensitivity leads to IGT^34, 43^. We calculated the product of these two variables known as the disposition index, which indicates the inability of the β-cell to compensate for increasing insulin resistance in Ob subjects with PD, who are at risk for T2D^29^. In our cohort of Ob children, we found the hyperbolic relationship between β-cell function and insulin sensitivity as described before in adults^12, 29^. The disposition index calculated by WBISI for insulin sensitivity as used in a previous study in adults^27^ and IGI for β-cell function showed clear separation and a ~ 50% reduction in Ob children with PD vs. nGT (Fig. 3). Similar to a previous study in adults^29^, the disposition index calculated by IGI and 1/fasting insulin was significantly different between nGT and PD Ob children; however, the magnitude of change was less than that of the WBISI and IGI disposition index. The lower disposition index in children with PD indicates that these subjects already have inadequate β-cell compensation for the degree of insulin resistance^37, 38^. This is also shown by their lower levels for ISSI-2, a marker for insulin resistance-adjusted β-cell function.

As an easy-to-calculate new marker for disordered glucose metabolism related to PD, we calculated the OGTT insulin 30-min/120-min ratio and observed that is unchanged in nGT Ob subjects relative to controls, yet it is significantly lower in subjects with PD. OGTT insulin at 30 min represents the early insulin response, whereas insulin at 120 min is mostly driven by insulin resistance. Healthy subjects will be able to secrete a larger amount of insulin in response to an oral glucose load within 30 min but need less insulin at 120 min to normalize the BG level. Higher insulin levels at 120 min vs. 30 min indicate a significant dysregulation of glucose metabolism. Instead of only measuring insulin at 120 min, the ratio has the advantage to correct for the higher insulin levels seen in pubertal vs. prepubertal subjects. In our study, the OGTT insulin ratio 30 min/120 min had considerable value in terms of sensitivity and specificity in order to detect PD (Table 3), particularly a higher sensitivity (88.9%) than the recommended HbA_1c_ threshold of 5.7% (sensitivity 23.5%) and even 5.4% (sensitivity 58.8%). Similar results were reported previously in adult subjects at risk for T2D, where individuals with PD showed significant delays in their insulin responses during an OGTT^44–46^ and significantly reduced early insulin responses^46^. Importantly, individuals with lasting late insulin response had a higher risk of developing T2D as established in a follow-up analysis^46^.

### Clinical implications

In several studies and current recommendations, it had been concluded that HbA_1c_ can identify children with PD with similar confidence as compared with fasting glucose and OGTT 2-h glucose^7, 8, 47^. However, studies investigating the specificity and sensitivity for identifying PD found only low predictable value for HbA_1c_, both in adults^48–50^ and in Ob children and adolescents^8, 9^. One contributing factor may be the use of different HbA_1c_ assays^51^ or ethnic differences in HbA_1c_ levels, with higher HbA_1c_ levels in African-American, Asian and Latino subjects^52, 53^. Other factors affecting HbA_1c_ levels are age, sex hormones, visceral fat distribution and genetic factors as reviewed elsewhere^54^. In our cohort of Caucasian youths, the recommended threshold for HbA_1c_ of 5.7% shows only low sensitivity to detect PD, which is similar to published results^7^. Brief fluctuations in glucose concentrations may not result in significant changes in HbA_1c_ levels^55, 56^, and more stringent threshold levels may be needed to identify risk for diabetes based on HbA_1c_ (Table 3)^7^.

Physiologically, glucose concentrations are regulated by insulin-dependent hepatic glucose output, whereas insulin levels reflect β-cell function in response to BG^57^. Although clamp studies, as well as intravenous glucose tolerance tests can result in more precise measures for β-cell function and insulin sensitivity, the OGTT is simpler to perform and can be used in large studies including children. Based on our results and in context of the existing literature discussed above, we recommend OGTT-derived measures for assessing the glucose metabolism, such as ISSI-2 and IGI for β-cell function and WBISI for insulin sensitivity. The OGTT disposition index using IGI and WBISI might be helpful in distinguishing between Ob subjects with and without decompensated glucose metabolism. Simple fasting surrogate markers for insulin sensitivity/resistance (QUICKI and HOMA-IR) are suitable for estimating the risk for PD and diabetes development, which correlate with clamp-derived measures^58^. Changes of these measures were already detectable. As outlined above, HbA_1c_ was not a sensitive marker that corresponds well to early metabolic changes leading to development disturbed glucose tolerance in our cohort. A longitudinal analysis will be necessary to confirm the predictive value of this marker. Other markers that also show low significance for the evaluation of the glucose metabolism included the FGIR, as it erroneously increased in subjects with increased glucose levels, and the HOMA-%B, as it accounts only for fasting insulin levels and does not present the dynamics of insulin secretion over time. Thus, HOMA-%B is more related to the degree of insulin resistance and does not seem to be suited to truly determine β-cell function^57^. During an OGTT, measuring insulin levels at 30 and 120 min and their ratio is a simple approach and might be a useful tool to identify decompensated glucose metabolism and PD.

The strength of our study is the examination and careful analysis of different markers of β-cell function and insulin sensitivity/resistance in children with and without PD. However, there are several limitations to our study, which need to be considered. The cross-sectional nature of our study prohibits us from determining the outcome of the baseline findings and does not allow conclusions about causality. Also, each subject might have a distinct risk for development of glucose intolerance, which might depend on genetic and epigenetic factors, as well as time course of obesity development and environmental exposures, which were not subject of this study. For assessment of obesity, only BMI percentiles and SDS-BMI were available but waist circumference was not measured. However, in a recent meta-analysis of 30 studies found no significant advantage of waist circumference over BMI in the prediction of cardiometabolic risk in children and adolescents^59^. Testing subjects by OGTT is limited by its poor reproducibility and results are dependent on other factors such as incretins and hepatic insulin extraction^57, 60^. We are also aware that the group of lean children is rather small. However, this group served only as a comparison group to estimate effects in lean children and most important statistical analyses were focused on the comparison of Ob children with vs. without PD. In addition, the tested indices are only surrogates for β-cell function and insulin sensitivity/resistance; however, previous studies showed that WBISI, QUICKI and HOMA-IR in particular correlate well with clamp-derived measures^14, 57, 58^. Finally, the insulin 30/120 ratio requires an OGTT and therefore cannot replace it. However, this ratio correlated negatively with OGTT-AUC_gluc0-120_ and might give some pathophysiological insight into β-cell dysfunction leading to glucose intolerance. Future studies and comparison with clamp studies are necessary to further establish this relatively simple parameter in the context of glucose metabolism disorder.

## Conclusions

In summary, several fasting and OGTT-derived measures, but not HbA_1c_, showed clear differences between Ob children with vs. without PD. Subjects with PD also showed a lower disposition index calculated by the insulinogenic index and WBISI as well as a lower ratio of OGTT insulin ratio at 30 min over 120 min, both measures need to be further established in future prospective studies.

## Electronic supplementary material

Suppl. Figure 1

## References

[1] Copeland KC (2011). Characteristics of adolescents and youth with recent-onset type 2 diabetes: the TODAY cohort at baseline. J. Clin. Endocrinol. Metab..

[2] Narasimhan S, Weinstock RS (2014). Youth-onset type 2 diabetes mellitus: lessons learned from the TODAY study. Mayo Clin. Proc..

[3] Badaru A, Pihoker C (2012). Type 2 diabetes in childhood: clinical characteristics and role of beta-cell autoimmunity. Curr. Diab. Rep..

[4] Dabelea D, Writing Group for the SfDiYSG (2007). Incidence of diabetes in youth in the United States. JAMA.

[5] May AL, Kuklina EV, Yoon PW (2012). Prevalence of cardiovascular disease risk factors among US adolescents, 1999-2008. Pediatrics..

[6] Gray S, Kim JK (2011). New insights into insulin resistance in the diabetic heart. Trends Endocrinol. Metab..

[7] American Diabetes A. (2017). 2. Classification and diagnosis of diabetes. Diabetes Care.

[8] Ehehalt S (2017). Diabetes screening in overweight and obese children and adolescents: choosing the right test. Eur. J. Pediatr..

[9] Nowicka P (2011). Utility of hemoglobin A(1c) for diagnosing prediabetes and diabetes in obese children and adolescents. Diabetes Care.

[10] Olson DE (2010). Screening for diabetes and pre-diabetes with proposed A1C-based diagnostic criteria. Diabetes Care.

[11] Heianza Y (2011). HbA1c 5.7-6.4% and impaired fasting plasma glucose for diagnosis of prediabetes and risk of progression to diabetes in Japan (TOPICS 3): a longitudinal cohort study. Lancet.

[12] Kahn SE (2003). The relative contributions of insulin resistance and beta-cell dysfunction to the pathophysiology of Type 2 diabetes. Diabetologia.

[13] Tam CS (2012). Defining insulin resistance from hyperinsulinemic-euglycemic clamps. Diabetes Care..

[14] Matsuda M, DeFronzo RA (1999). Insulin sensitivity indices obtained from oral glucose tolerance testing: comparison with the euglycemic insulin clamp. Diabetes Care.

[15] Henderson M (2011). Measuring insulin sensitivity in youth: how do the different indices compare with the gold-standard method?. Diabetes Metab..

[16] Singh B, Saxena A (2010). Surrogate markers of insulin resistance: a review. World J. Diabetes.

[17] Lee SH (2015). Changes in metabolic health status over time and risk of developing type 2 diabetes: a prospective cohort study. Medicine (Baltimore).

[18] Guerrero-Romero F (2010). The product of triglycerides and glucose, a simple measure of insulin sensitivity. Comparison with the euglycemic-hyperinsulinemic clamp. J. Clin. Endocrinol. Metab..

[19] Moss A, Kunze D, Wabitsch M (2011). [Evidence-based therapy guideline of the German Working Group on Obesity in Childhood and Adolescence]. Bundesgesundheitsblatt Gesundheitsforschung Gesundheitsschutz.

[20] Reinehr T, de Sousa G, Toschke AM, Andler W (2006). Long-term follow-up of cardiovascular disease risk factors in children after an obesity intervention. Am. J. Clin. Nutr..

[21] Cole TJ (1990). The LMS method for constructing normalized growth standards. Eur. J. Clin. Nutr..

[22] Marshall WA, Tanner JM (1969). Variations in pattern of pubertal changes in girls. Arch. Dis. Child..

[23] Marshall WA, Tanner JM (1970). Variations in the pattern of pubertal changes in boys. Arch. Dis. Child..

[24] Wang XM, Jiang YJ, Liang L, Du LZ (2008). Changes of ghrelin following oral glucose tolerance test in obese children with insulin resistance. World J. Gastroenterol..

[25] Uwaifo G. I. et al. Indices of insulin action, disposal, and secretion derived from fasting samples and clamps in normal glucose-tolerant black and white children. *Diabetes Care* **25**, 2081–2087 (2002). 10.2337/diacare.25.11.2081 12401760

[26] Matthews DR (1985). Homeostasis model assessment: insulin resistance and beta-cell function from fasting plasma glucose and insulin concentrations in man. Diabetologia.

[27] Retnakaran R (2008). Hyperbolic relationship between insulin secretion and sensitivity on oral glucose tolerance test. Obesity (Silver Spring).

[28] Retnakaran R, Qi Y, Goran MI, Hamilton JK (2009). Evaluation of proposed oral disposition index measures in relation to the actual disposition index. Diabet. Med..

[29] Utzschneider KM (2009). Oral disposition index predicts the development of future diabetes above and beyond fasting and 2-h glucose levels. Diabetes Care.

[30] Yeckel CW (2004). Validation of insulin sensitivity indices from oral glucose tolerance test parameters in obese children and adolescents. J. Clin. Endocrinol. Metab..

[31] Allard P (2003). Distribution of fasting plasma insulin, free fatty acids, and glucose concentrations and of homeostasis model assessment of insulin resistance in a representative sample of Quebec children and adolescents. Clin. Chem..

[32] Sinha R (2002). Prevalence of impaired glucose tolerance among children and adolescents with marked obesity. N. Engl. J. Med..

[33] Michaliszyn SF (2014). beta-cell function, incretin effect, and incretin hormones in obese youth along the span of glucose tolerance from normal to prediabetes to type 2 diabetes. Diabetes.

[34] Weiss R (2005). Beta-cell function across the spectrum of glucose tolerance in obese youth. Diabetes.

[35] Goran MI (2004). Impaired glucose tolerance and reduced beta-cell function in overweight Latino children with a positive family history for type 2 diabetes. J. Clin. Endocrinol. Metab..

[36] Nolfe G, Spreghini MR, Sforza RW, Morino G, Manco M (2012). Beyond the morphology of the glucose curve following an oral glucose tolerance test in obese youth. Eur. J. Endocrinol..

[37] Yeckel CW (2005). The normal glucose tolerance continuum in obese youth: evidence for impairment in beta-cell function independent of insulin resistance. J. Clin. Endocrinol. Metab..

[38] Bacha F, Lee S, Gungor N, Arslanian SA (2010). From pre-diabetes to type 2 diabetes in obese youth: pathophysiological characteristics along the spectrum of glucose dysregulation. Diabetes Care.

[39] Juonala M (2011). Childhood adiposity, adult adiposity, and cardiovascular risk factors. N. Engl. J. Med..

[40] Bao W, Srinivasan SR, Berenson GS (1996). Persistent elevation of plasma insulin levels is associated with increased cardiovascular risk in children and young adults. The Bogalusa Heart Study. Circulation.

[41] Nakanishi S, Yoneda M, Maeda S (2013). Impact of glucose excursion and mean glucose concentration in oral glucose-tolerance test on oxidative stress among Japanese Americans. Diabetes Metab. Syndr. Obes.

[42] DeFronzo RA, Tobin JD, Andres R (1979). Glucose clamp technique: a method for quantifying insulin secretion and resistance. Am. J. Physiol..

[43] Cali AM, Bonadonna RC, Trombetta M, Weiss R, Caprio S (2008). Metabolic abnormalities underlying the different prediabetic phenotypes in obese adolescents. J. Clin. Endocrinol. Metab..

[44] Abdul-Ghani MA, Jenkinson CP, Richardson DK, Tripathy D, DeFronzo RA (2006). Insulin secretion and action in subjects with impaired fasting glucose and impaired glucose tolerance: results from the Veterans administration genetic epidemiology study. Diabetes.

[45] Hanefeld M (2003). Insulin secretion and insulin sensitivity pattern is different in isolated impaired glucose tolerance and impaired fasting glucose: the risk factor in Impaired Glucose Tolerance for Atherosclerosis and Diabetes study. Diabetes Care.

[46] Hayashi T (2013). Patterns of insulin concentration during the OGTT predict the risk of type 2 diabetes in Japanese Americans. Diabetes Care.

[47] Lee AM, Fermin CR, Filipp SL, Gurka MJ, DeBoer MD (2017). Examining trends in prediabetes and its relationship with the metabolic syndrome in US adolescents, 1999-2014. Acta. Diabetol..

[48] Bianchi C (2012). Pathogenetic mechanisms and cardiovascular risk: differences between HbA(1c) and oral glucose tolerance test for the diagnosis of glucose tolerance. Diabetes Care.

[49] Lipska KJ (2010). Identifying dysglycemic states in older adults: implications of the emerging use of hemoglobin A1c. J. Clin. Endocrinol. Metab..

[50] Vega-Vazquez M. A., Ramirez-Vick M., Munoz-Torres F. J., Gonzalez-Rodriguez L. A., Joshipura K., Comparing glucose and hemoglobin A1c diagnostic tests among a high metabolic risk Hispanic population. *Diabetes Metab. Res. Rev*. **33**, 1–14 (2017). 10.1002/dmrr.2874 PMC5413375 27933750

[51] Chan CL (2014). Hemoglobin A1c assay variations and implications for diabetes screening in obese youth. Pediatr. Diabetes.

[52] Chapp-Jumbo E, Edeoga C, Wan J, Dagogo-Jack S (2012). Pathobiology of Prediabetes in a Biracial Cohort Research G. Ethnic disparity in hemoglobin A1c levels among normoglycemic offspring of parents with type 2 diabetes mellitus. Endocr. Pract..

[53] Cavagnolli G, Pimentel AL, Freitas PA, Gross JL, Camargo JL (2017). Effect of ethnicity on HbA1c levels in individuals without diabetes: systematic review and meta-analysis. PLoS ONE.

[54] Makris K, Spanou L (2011). Is there a relationship between mean blood glucose and glycated hemoglobin?. J. Diabetes Sci. Technol..

[55] Ulf S, Ragnar H, Arne WP, Johnny L (2008). Do high blood glucose peaks contribute to higher HbA1c? Results from repeated continuous glucose measurements in children. World J. Pediatr..

[56] Kilpatrick ES (2000). Glycated haemoglobin in the year 2000. J. Clin. Pathol..

[57] Muniyappa R, Lee S, Chen H, Quon MJ (2008). Current approaches for assessing insulin sensitivity and resistance in vivo: advantages, limitations, and appropriate usage. Am. J. Physiol. Endocrinol. Metab..

[58] Conwell LS, Trost SG, Brown WJ, Batch JA (2004). Indexes of insulin resistance and secretion in obese children and adolescents: a validation study. Diabetes Care.

[59] Lo K., Wong M., Khalechelvam P., Tam W., Waist-to-height ratio, body mass index and waist circumference for screening paediatric cardio-metabolic risk factors: a meta-analysis.*Obes. Rev.* **17**, 1258–1275 (2016). 10.1111/obr.12456 27452904

[60] Mari A, Pacini G, Murphy E, Ludvik B, Nolan JJ (2001). A model-based method for assessing insulin sensitivity from the oral glucose tolerance test. Diabetes Care.

