# Supplementary material for: Assessment of disturbed glucose metabolism and surrogate measures of insulin sensitivity in obese children and adolescents
Source: Nutr Diabetes. 2017 Dec 14;7(12):301. doi: 10.1038/s41387-017-0004-y (PMC5865547; doi:10.1038/s41387-017-0004-y)
Supplement: Supplementary file 1 — Suppl. Figure 1 [file 41387_2017_4_MOESM1_ESM.pptx]

## Slide 1
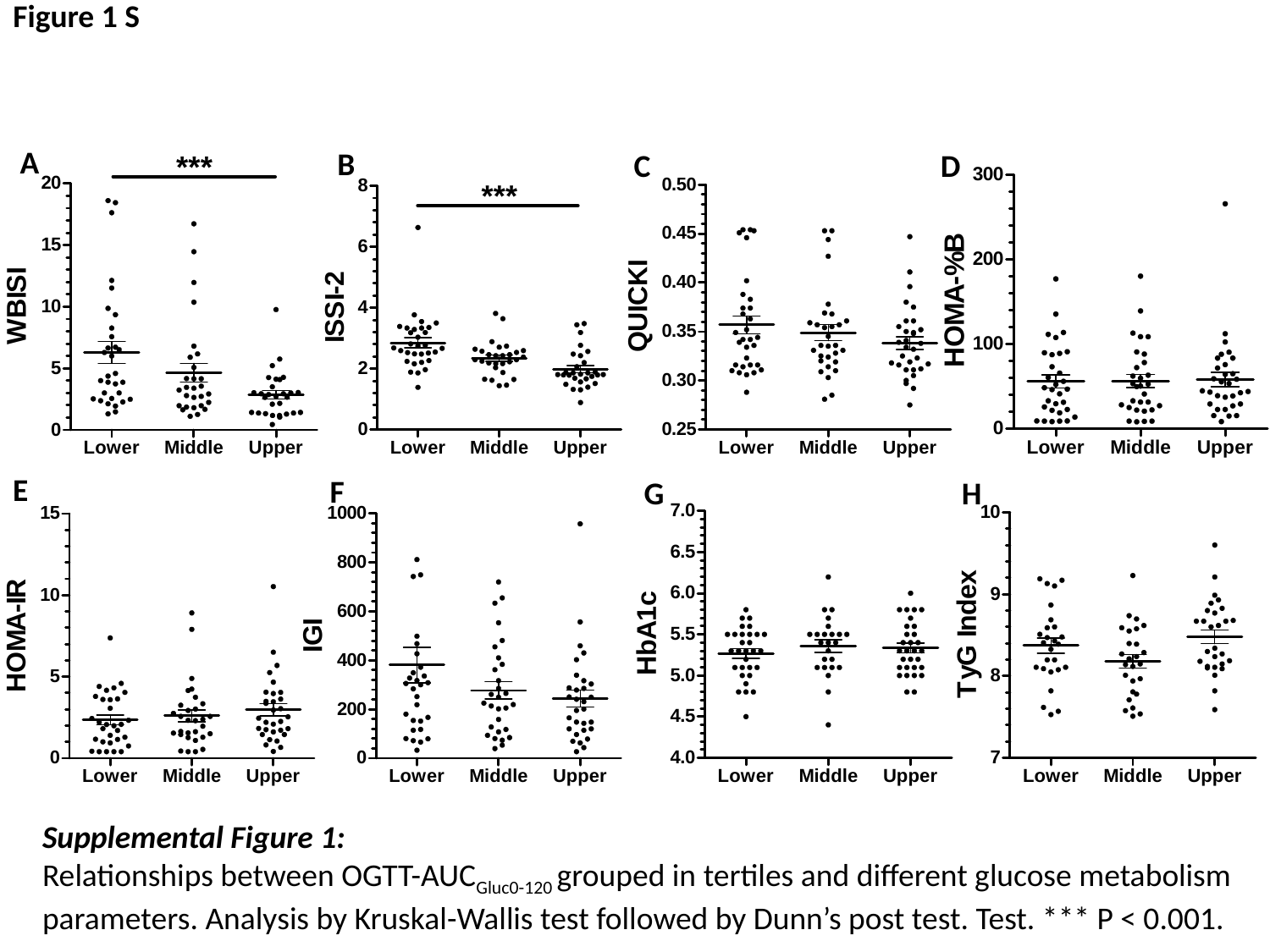

Figure 1 S
A
B
C
D
E
F
G
H
Supplemental Figure 1:
Relationships between OGTT-AUCGluc0-120 grouped in tertiles and different glucose metabolism parameters. Analysis by Kruskal-Wallis test followed by Dunn’s post test. Test. *** P < 0.001.
